# Impact of race‐based customization on detection of fetal growth restriction

**DOI:** 10.1002/uog.70096

**Published:** 2025-09-05

**Authors:** P. Ramesh, L. Lemon, J. C. Larkin

**Affiliations:** ^1^ Department of Obstetrics, Gynecology and Reproductive Sciences University of Pittsburgh School of Medicine Pittsburgh PA USA

**Keywords:** customized growth curve, fetal growth restriction, growth standard, Hadlock, obstetric racial disparity, small‐for‐gestational age

## Abstract

**Objective:**

Fetal growth standards determine which fetuses are diagnosed with fetal growth restriction (FGR) and become candidates for enhanced fetal monitoring. Given the existence of race‐based differences in fetal and neonatal weights, we sought to determine the impact of race‐based customization of fetal growth curves on the antenatal detection of FGR.

**Methods:**

This was a retrospective cohort study of 8731 individuals who identified as either White or Black and delivered a liveborn singleton at Magee‐Womens Hospital (MWH), Pittsburgh, PA, USA, between January 2003 and January 2013, with at least one sonographic measurement of estimated fetal weight (EFW) taken at 23–41 weeks' gestation. We compared the rates of antenatal FGR diagnosis when EFW was assessed using three distinct growth standards: (1) a standard used at MWH from 2012 to 2018, customized based on the height, weight, parity and race of the pregnant individual (Cust‐Race); (2) the same standard without adjustment for race (Cust‐NoRace); and (3) the Hadlock standard. Analyses were stratified by the race of the pregnant individual and classification of the neonate as small‐for‐gestational age (SGA) based on birth weight < 10^th^ percentile.

**Results:**

The study population included 1458 (16.7%) individuals who self‐identified as Black and 7273 (83.3%) who self‐identified as White. SGA was diagnosed in 663 (7.6%) newborns, and was significantly more common in those born to Black *vs* White individuals (172/1458 (11.8%) *vs* 491/7273 (6.8%); *P* < 0.001). Among SGA newborns, 286 (43.1%) had at least one antenatal ultrasound scan that met the diagnostic criteria for FGR using the Cust‐Race standard, compared with 306 (46.2%) using Cust‐NoRace and 335 (50.5%) using Hadlock; only the difference in FGR diagnosis rate between Cust‐Race and Hadlock was significant (*P* = 0.007). For newborns of Black individuals who were SGA at birth, the Cust‐Race growth standard diagnosed 52 (30.2%) cases of antenatal FGR, compared with 72 (41.9%) for Cust‐NoRace and 77 (44.8%) for Hadlock; again, only the difference in FGR diagnosis rate between Cust‐Race and Hadlock was significant (*P* = 0.005). The antenatal detection of FGR among newborns of White individuals who were SGA at birth was similar across standards, with 234 (47.7%) detected by Cust‐Race, 234 (47.7%) by Cust‐NoRace and 258 (52.5%) by Hadlock.

**Conclusions:**

Customization of growth standards with a race variable did not improve the antenatal detection of FGR compared with the Hadlock standard. The Hadlock standard demonstrated an improved ability to detect FGR among Black patients without a negative effect on White patients. Moving away from race‐specific growth standards may help to eliminate inequities in resource allocation and reduce racial disparities in obstetric care. © 2025 The Author(s). *Ultrasound in Obstetrics & Gynecology* published by John Wiley & Sons Ltd on behalf of International Society of Ultrasound in Obstetrics and Gynecology.

## INTRODUCTION

Fetal growth restriction (FGR) is associated with fetal death and neonatal morbidity and mortality^1^, ^2^. The antenatal diagnosis of FGR prompts enhanced fetal monitoring, including umbilical artery Doppler studies, intended to mitigate these adverse outcomes. Thus, the decision to use a specific fetal growth standard to diagnose FGR drives resource allocation and determines which fetuses undergo additional monitoring. While there is agreement among several professional societies in defining FGR as estimated fetal weight (EFW) or abdominal circumference < 10^th^ percentile^3^, limited consensus exists on the optimal growth standard used to delineate these thresholds. Variation in the methods and reference populations used to generate growth standards contributes to widespread inconsistency in FGR diagnostic criteria across institutions.

Non‐Hispanic Black Americans experience disproportionately high rates of adverse obstetric and perinatal outcomes; their rates of fetal and infant mortality are more than double those of non‐Hispanic White individuals^4^, ^5^, ^6^, ^7^. With the recognition that elements of structural racism, such as income and neighborhood segregation, negatively impact obstetric outcome, it is imperative to recognize that racism, not race, drives race‐based disparities in adverse obstetric outcome^5^, ^8^. Race‐based differences in both fetal and neonatal weights have been well documented^4^. Specifically, at comparable gestational ages, fetuses and neonates born to non‐Hispanic Black individuals weigh less, on average, compared with those born to non‐Hispanic White individuals^9^. Approaches to account for these differences include stratifying growth standards by race or customizing them based on the characteristics of the pregnant individual. In contrast, certain fetal growth standards are applied universally.

In the light of race‐based differences in both fetal growth and adverse perinatal outcome, we sought to assess the impact of race‐based customization of fetal growth standards on the diagnosis of FGR.

## METHODS

This retrospective cohort study analyzed 9378 pregnant individuals who delivered a liveborn singleton at Magee‐Womens Hospital (MWH), Pittsburgh, PA, USA, between January 2003 and January 2013. The study was approved by the University of Pittsburgh Institutional Review Board (protocol 20 110 406). All included patients had at least one sonographic EFW assessment at 23–41 weeks' gestation. Owing to limited sample sizes in other racial subgroups (*n* = 647), our analysis focused on pregnant individuals who self‐identified as Black or White (*n* = 8731). EFW was calculated using the Hadlock‐III formula, incorporating head circumference, abdominal circumference and femur length^10^.

The EFW from each ultrasound scan was assessed using three fetal weight standards. The first growth standard, Cust‐Race (CR), was derived based on methods established by Gardosi *et al*.^11^, ^12^, ^13^, ^14^, ^15^, ^16^, independently of and prior to the current analysis. This standard was customized according to the height, weight, parity and race of the pregnant individual. It was developed using MWH delivery data and was used at MWH between 2012 and 2018. The CR formula to calculate a term optimal weight (TOW) in g at 40 weeks is as follows:

CR‐TOW = 3477.4 + 7.277 × (weight (kg) – 64) – 0.058 × (weight (kg) – 64)^2^ + 5.707 × (height (cm) – 163) + (90.766 (if parity = 1) or + 116.144 (if parity = 2) or + 107.693 (if parity ≥ 3)) – 155.869 (if self‐identifies as Black or of African descent).

The second growth standard, Cust‐NoRace (CNR), used the same algorithm to calculate TOW but excluded the final term adjusting for race. The threshold for diagnosing FGR at 40 weeks (FGR_40_) for both the CR and CNR models was then calculated based on a 0.1135 coefficient of variation and *Z*‐score for the 10^th^ percentile of –1.282:

FGR_40_ = TOW – (TOW × 0.1135 × 1.282)

The diagnostic threshold for FGR at other gestational ages was extrapolated based on the growth trajectories of Hadlock *et al*.^17^. This method of calculating a customized TOW and FGR threshold at 40 weeks, followed by extrapolation for all gestational ages using the Hadlock trajectory, is consistent with the methods of Gardosi *et al*.^11^, ^12^, ^13^.

The third growth standard, Hadlock (HAD), was the sonographically derived fetal weight standard published by Hadlock *et al*.^17^. The HAD standard is applied consistently for all patients without adjustment for race or other variables. FGR was diagnosed when the sonographic EFW was < 10^th^ percentile.

Neonates were characterized as small‐for‐gestational age (SGA) if the birth weight was < 10^th^ percentile on the Fenton scale, as used by neonatologists at MWH^18^. The Fenton scale is based exclusively on gestational age at birth and fetal sex, and is not modified by race or ethnicity. We calculated the frequency of FGR diagnosis on at least one ultrasound scan during pregnancy in the total population and in those who were and those who were not characterized as SGA at birth, with and without race‐based stratification.

Demographic and pregnancy characteristics were compared using Student's *t*‐test and the chi‐square test for continuous and categorical variables, respectively. We compared the rates of FGR diagnosis according to each growth standard using the two‐proportion *Z*‐test. Statistical significance was set at a two‐tailed *P* of 0.05; for comparisons involving three groups, the Bonferroni‐corrected *P* of 0.0167 was used.

## RESULTS

The study population comprised 1458 (16.7%) individuals who identified as Black and 7273 (83.3%) who identified as White. Demographic and pregnancy characteristics differed significantly by race (Table 1). On average, Black pregnant individuals were younger than White individuals (mean ± SD, 23.5 ± 6.0 years *vs* 29.5 ± 5.9 years) and had lower educational attainment. Compared with White pregnant individuals, Black individuals had higher rates of prepregnancy obesity (26.5% *vs* 22.0%) and parity ≥ 3 (9.3% *vs* 5.7%). There was no significant racial difference in the rate of preterm birth.

**Table 1:** Demographic and pregnancy characteristics of study population, according to race of pregnant individual

Characteristic	Total (*n* = 8731)	White (*n* = 7273)	Black (*n* = 1458)	*P* *
Age (years)	28.5 ± 6.3	29.5 ± 5.9	23.5 ± 6.0	< 0.001
Prepregnancy BMI				< 0.001
Underweight (< 18.5 kg/m^2^)	281 (3.2)	235 (3.2)	46 (3.2)	
Normal (18.5–24.9 kg/m^2^)	4559 (52.2)	3886 (53.4)	673 (46.2)	
Overweight (25.0–29.9 kg/m^2^)	1906 (21.8)	1554 (21.4)	352 (24.1)	
Obese (≥ 30.0 kg/m^2^)	1985 (22.7)	1598 (22.0)	387 (26.5)	
Education				< 0.001
Less than high school	842 (9.6)	467 (6.4)	375 (25.7)	
High school	1951 (22.3)	1412 (19.4)	539 (37.0)	
Partial college	2014 (23.1)	1651 (22.7)	363 (24.9)	
College or higher	3842 (44.0)	3671 (50.5)	171 (11.7)	
Unknown	82 (0.9)	72 (1.0)	10 (0.7)	
Parity				< 0.001
0	4317 (49.4)	3568 (49.1)	749 (51.4)	
1	2703 (31.0)	2334 (32.1)	369 (25.3)	
2	1161 (13.3)	957 (13.2)	204 (14.0)	
≥ 3	550 (6.3)	414 (5.7)	136 (9.3)	
GA at delivery				0.268
< 37 weeks	1187 (13.6)	1002 (13.8)	185 (12.7)	
≥ 37 weeks	7544 (86.4)	6271 (86.2)	1273 (87.3)	
SGA†	663 (7.6)	491 (6.8)	172 (11.8)	< 0.001

Data are given as mean ± SD or *n* (%).

* Student's *t*‐test or chi‐square test, as appropriate.

† Defined as birth weight < 10^th^ percentile. BMI, body mass index; GA, gestational age; SGA, small‐for‐gestational age.

According to the Fenton standard, 7.6% (*n* = 663) of neonates were SGA at birth. SGA at birth was significantly more frequent among neonates born to Black individuals (172/1458 (11.8%)) compared with those born to White individuals (491/7273 (6.8%)) (*P* < 0.001).

Table 2 presents the rate of antenatal FGR diagnosis according to the three growth standards and stratified by race. In the overall cohort, the rate of FGR diagnosis was similar across the three growth standards: 7.7% by the CR standard, 8.6% by the CNR standard and 8.3% by the HAD standard. After applying Bonferroni correction (significance threshold of *P* < 0.0167), none of these differences reached statistical significance. Among fetuses of White individuals (*n* = 7273), the rate of FGR was nearly identical across the three growth standards. By contrast, fetuses of Black individuals (*n* = 1458) had a significantly lower rate of FGR using the CR standard (6.9%) compared with CNR (12.5%) and HAD (11.0%). Moreover, when comparing the rate of FGR diagnosis between Black and White individuals, there were significant differences using the CNR and HAD standards.

**Table 2:** Rate of fetal growth restriction (FGR) diagnosis according to Cust‐Race (CR), Cust‐NoRace (CNR) and Hadlock (HAD) growth standards, stratified by birth weight and race of pregnant individual

		Rate of FGR (*n* (%))					
Group	*n*	CR	CNR	HAD	*P*_CR *vs* CNR_	*P*_CR *vs* HAD_	*P*_CNR *vs* HAD_
All	8731	672 (7.7)	751 (8.6)	724 (8.3)	0.03	0.13	0.46
White	7273	567 (7.8)	567 (7.8)	560 (7.7)	> 0.99	0.85	0.85
Black	1458	101 (6.9)	182 (12.5)	160 (11.0)	< 0.001	< 0.001	0.23
SGA*	663	286 (43.1)	306 (46.2)	335 (50.5)	0.27	0.007	0.11
White	491	234 (47.7)	234 (47.7)	258 (52.5)	> 0.99	0.13	0.13
Black	172	52 (30.2)	72 (41.9)	77 (44.8)	0.02	0.005	0.59
Non‐SGA	8068	379 (4.7)	444 (5.5)	387 (4.8)	0.03	0.85	0.05
White	6782	332 (4.9)	332 (4.9)	305 (4.5)	> 0.99	0.22	0.22
Black	1286	49 (3.8)	111 (8.6)	84 (6.5)	< 0.001	0.002	0.05

* Defined as birth weight < 10^th^ percentile. SGA, small‐for‐gestational age.

**Figure 1:**
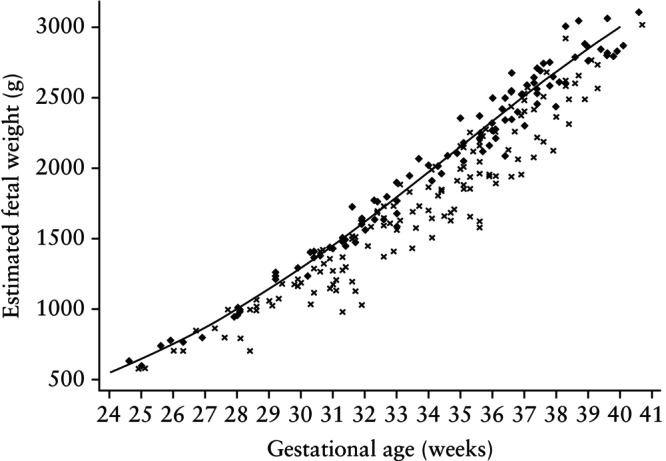
Estimated fetal weight (EFW) throughout gestation in Black individuals who met diagnostic criteria for fetal growth restriction according to both Cust‐NoRace and Cust‐Race growth standards (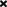) or Cust‐NoRace standard only (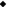). Line indicates 10^th^ percentile of EFW using Hadlock growth standard.

Figure 1 shows the EFW and gestational age at ultrasound assessment for all fetuses of Black individuals that met the diagnostic criteria for FGR according to both the CR and CNR standards or the CNR standard only. This graph demonstrates that, at a given gestational age, a Black fetus had to have a lower EFW to be diagnosed with FGR by the CR standard. By design, all fetuses of White individuals that met the diagnostic criteria for FGR according to the CR standard were also diagnosed with FGR by the CNR standard.

We next sought to compare the rate of FGR according to each standard specifically for fetuses classified as SGA at birth (*n* = 663). In general, antenatal FGR detection in this population was limited across all standards. Among SGA newborns, 286 (43.1%) were diagnosed with FGR using the CR standard, compared with 306 (46.2%) using CNR and 335 (50.5%) using HAD (Table 2). The only statistically significant difference was between CR and HAD. When stratified by race, antenatal detection of FGR among SGA newborns of White individuals (*n* = 491) did not differ significantly across the three growth standards: 47.7% by the CR and CNR standards, and 52.5% by the HAD standard. By contrast, among SGA newborns of Black individuals, the CR standard diagnosed significantly fewer cases of antenatal FGR compared with the HAD standard (52/172 (30.2%) *vs* 77/172 (44.8%)). The rate of antenatal FGR detection among SGA newborns of Black individuals did not differ significantly between the CR *vs* CNR standards or between the CNR *vs* HAD standards.

Finally, to evaluate for overdiagnosis or misdiagnosis of FGR related to overly permissive diagnostic criteria, we compared the rate of antenatal FGR diagnosis among fetuses that were not SGA at birth (*n* = 8068) according to each growth standard. None of these comparisons was statistically significant, with similarly low rates of FGR diagnosis across the three growth standards (Table 2). However, race‐stratified analysis demonstrated differential rates of FGR diagnosis between the growth standards among fetuses of Black individuals that were not SGA at birth (*n* = 1286). In this population, the CR standard yielded significantly fewer FGR diagnoses compared with CNR and HAD.

## DISCUSSION

We compared three distinct fetal growth standards in the antenatal detection of FGR and the prediction of SGA status at birth. In the overall study population, evaluated without race‐based stratification, a customized, race‐based growth curve was less likely to identify FGR in fetuses that were SGA at birth compared with the Hadlock standard. This trend persisted in SGA newborns of Black individuals when race‐based stratification was applied. Customization with a race variable did result in lower rates of FGR diagnosis among fetuses of Black individuals that were not SGA at birth. However, overall, customization appeared to add little value in the antenatal diagnosis of FGR.

Detection of FGR among fetuses that were SGA at birth was poor, with only the HAD standard diagnosing over 50% of cases antenatally. Given that the HAD standard outperformed the race‐customized standard, our data suggest that the effort and potential confusion associated with implementing customization offers little benefit. Although the study population of Hadlock *et al*.^17^ was predominantly White and middle class, their growth curve is applied consistently, irrespective of race, socioeconomic status and ethnicity. In our study, use of the HAD standard resulted in increased diagnosis of FGR among Black fetuses that were not SGA at birth compared with the CR standard. However, the improvement in detection of SGA at birth compared with the CR standard (44.8% *vs* 30.2%) is much greater than the corresponding decline in accurate identification of non‐SGA fetuses (93.5% *vs* 96.2%). It is likely that the consistent application of the HAD standard to all fetuses contributes to a reduction in race‐based disparities in the detection of FGR. Given that the antenatal diagnosis of FGR results in enhanced Doppler monitoring, which is known to improve fetal outcome, moving away from customized growth standards may promote equity in resource allocation and clinical outcomes^19^, ^20^, ^21^.

Conflicting data exist regarding the utility of customized growth curves, as prior evidence suggests that they may help to predict adverse outcome and reduce healthcare disparity^22^. Customized curves, such as those developed by the National Institute of Child Health and Human Development (NICHD) fetal growth studies, aim to address possible differences between fetuses of various racial and ethnic backgrounds^23^. In some studies, the curve of Hadlock *et al*.^17^ has been shown to outperform the NICHD standards and is comparable to the population‐based World Health Organization and INTERGROWTH‐21^st^ standards in the prediction of SGA newborns^24^, ^25^. Other studies acknowledge the limitations of any growth standard, although they suggest that customized curves may predict perinatal morbidity of the highest‐risk groups more reliably compared with population‐based standards^26^, ^27^.

It may be argued that the fetuses of Black individuals not identified as FGR by the CR standard but identified as such by the CNR or HAD standards may be appropriately omitted because they are ‘physiologically small’. However, outcomes‐based analysis suggest that, at comparable fetal weights, rates of adverse outcomes are higher for fetuses and newborns of Black people compared with other racial/ethnic groups^28^, ^29^. While our study is informative in relation to the accuracy of FGR diagnosis among SGA newborns, further outcomes‐based analysis, using both customized and uncustomized standards, would shed more light on the utility of specific fetal growth standards. Future research should also focus on the identification of other contributors to structural racism that perpetuate healthcare disparities, as it is now well accepted that systemic policies drive race‐based differences in a host of healthcare outcomes^8^, ^30^.

Our study has several strengths. All subjects underwent ultrasound scans and gave birth at our institution, allowing for comparisons based on ultrasound findings and birth‐weight parameters. The study reflects actual patterns of clinical care; all ultrasound scans were obtained for a clinical indication and interpreted by a consistent group of providers, and the resulting management was by standard practice outside of a research protocol.

Several limitations should also be acknowledged. FGR was diagnosed by ultrasonographic EFW < 10^th^ percentile only, but it can also be diagnosed by an abdominal circumference < 10^th^ percentile. We chose to exclude the latter ultrasound finding given a lack of established growth trajectories for abdominal circumference and no postnatal values for comparison. All ultrasound assessments were clinically indicated, so our findings may not be applicable to universal growth ultrasound scans. Additionally, we did not evaluate perinatal outcomes other than birth weight.

Moreover, the customized TOW formula was developed prior to our analysis. Because the original dataset used to derive the model was not available, we were unable to generate a model without the inclusion of race as a covariate. Thus, the CNR model essentially assessed fetuses of White individuals according to the CR standard. It should be noted that we do not propose that the CNR standard be applied clinically. Our intention was simply to demonstrate the impact of race‐based customization compared with universal application of growth standards.

Lastly, the demographic distribution of our study cohort reflects our local population, which limits the generalizability of our findings. Our ability to perform meaningful analysis of individuals that did not identify as either White or Black was limited by the small sample size for each individual race. However, we contend that the principles uncovered by our findings reflect the national consequences of systemic racism. In our population, Black pregnant individuals were younger, with lower educational attainment, and were more likely to have higher parity and prepregnancy obesity. These are all markers associated with structural racism^31^. As discussed by Belfort *et al*.^32^, by using race‐specific growth standards clinicians may overlook social determinants of health contributing to racial disparities. In recognition of such biases, there is a growing trend to do away with race‐based differences throughout medicine, including in clinical indices such as glomerular filtration rate and hemoglobin^33^, ^34^. Race is no longer used in predicting the success of a vaginal birth after Cesarean section^35^; we argue that similar practice should be adopted in other obstetric areas.

Fetuses of Black pregnant people, in the United States of America, are at higher risk of adverse outcome compared with those of White individuals^28^. Race‐based customization of fetal growth standards potentially normalizes pathological FGR in fetuses of Black pregnant people. The underdiagnosis of FGR will contribute to missed opportunities for beneficial interventions, such as umbilical artery Doppler assessment, antenatal surveillance and administration of antenatal corticosteroids. A more stringent threshold for diagnosing FGR in fetuses of Black individuals resulting from race‐based customization likely leads to a systemic misappropriation of resources that exacerbates racial disparities in fetal and neonatal outcomes. Given this trend, we urge clinicians to recognize that race‐based customized growth curves may perpetuate systemic racism in obstetric care.

## Data Availability

The data that support the findings of this study are available on request from the corresponding author. The data are not publicly available due to privacy or ethical restrictions.
